# The Adjuvanted Recombinant Zoster Vaccine Confers Long-Term Protection Against Herpes Zoster: Interim Results of an Extension Study of the Pivotal Phase 3 Clinical Trials ZOE-50 and ZOE-70

**DOI:** 10.1093/cid/ciab629

**Published:** 2021-07-20

**Authors:** Céline Boutry, Andrew Hastie, Javier Diez-Domingo, Juan Carlos Tinoco, Chong-Jen Yu, Charles Andrews, Jean Beytout, Covadonga Caso, Huey-Shinn Cheng, Hee Jin Cheong, Eun Ju Choo, Dan Curiac, Emmanuel Di Paolo, Marc Dionne, Tamara Eckermann, Meral Esen, Murdo Ferguson, Wayne Ghesquiere, Shinn-Jang Hwang, Thiago Junqueira Avelino-Silva, Pavel Kosina, Chiu-Shong Liu, Jukka Markkula, Beate Moeckesch, Cláudia Murta de Oliveira, Dae Won Park, Karlis Pauksens, Paola Pirrotta, Georg Plassmann, Carol Pretswell, Lars Rombo, Bruno Salaun, Johan Sanmartin Berglund, Isabelle Schenkenberger, Tino Schwarz, Meng Shi, Benita Ukkonen, Toufik Zahaf, Cristiano Zerbini, Anne Schuind, Anthony L Cunningham, Zoster-049 Study Group, Michael Adams, Anitta Ahonen, Eugene Athan, Jose-Fernando Barba-Gómez, Piero Barbanti, Elisabeth Barberan, Alain Baty, Niklas Bengtsson, Juergen Berger-Roscher, Katarina Berndtsson Blom, Loïc Boucher, Alain Boye, François Brault, Laurie Breger, Carles Brotons Cuixart, Christine Cerna, Clóvis Cunha, Benoit Daguzan, Antje Dahmen, Susan Datta, Maria Giuseppina Desole, Petr Dite, Jan Dutz, John Earl, William Ellison, Jurij Eremenko, Takashi Eto, Xavier Farrés Fabré, Cecil Farrington, Pierre André Ferrand, Matthew Finneran, David Francyk, Marshall Freedman, George Freeman, Peter Gal, Jean-Sebastien Gauthier, Beatrice Gerlach, Iris Gorfinkel, Christine Grigat, Josef Grosskopf, Monika Hamann, Pascal Hanrion, Paul Hartley, Ken Heaton, Agnes Himpel-Boenninghoff, Thomas Horacek, David Shu Cheong Hui, Yieng Huong, Giancarlo Icardi, Gabriele Illies, Junya Irimajiri, Alen Jambrecina, Hyo Youl Kim, Christiane Klein, Uwe Kleinecke, Hans-Joachim Koenig, Satu Kokko, Pekka Koskinen, Maximilian Kropp, Rie Kuroki, Outi Laajalahti, Pierre Lachance, Jacob Lee, Jin-Soo Lee, Peter Levins, Robert Lipetz, Bo Liu, Martin Lundvall, Mary Beth Manning, Frederick Martin, Pyrene Martínez Piera, Damien McNally, Shelly McNeil, Guglielmo Migliorino, Stephan Morscher, Michael Mueller, Abul Kashem Munir, Kenjiro Nakamura, Silvia Narejos Pérez, Yuji Naritomi, Patrice Nault, José Luiz Neto, Concepción Núñez López, Hiroaki Ogata, Åke Olsson, Pauliina Paavola, Janice Patrick, Mercè Pérez Vera, Airi Poder, Terry Poling, Samir Purnell-Mullick, George Raad, Michael Redmond, Philippe Remaud, Ernie Riffer, Patrick Robert, Alex Rodríguez Badia, Maria Luisa Rodríguez de la Pinta, Robert Rosen, Shari Rozen, Dominique Saillard, Joachim Sauter, Axel Schaefer, Juergen Schmidt, Bernhard Schmitt, Christian Schubert, Ilkka Seppa, Edmund Kwok Yiu Sha, Gerald Shockey, Sylvia Shoffner, Elina Sirnela-Rif, Tommaso Staniscia, Hirohiko Sueki, Shin Suzuki, Denis Taminau, Guy Tellier, Manuel Terns Riera, Azhar Toma, Nicole Toursarkissian, Mark Turner, Anna Vilella Morató, Juergen Wachter, Brian Webster, Karl Wilhelm, Jonathan Wilson, Wilfred Yeo, Irina Zahharova

**Affiliations:** 1 Aixial, an Alten Company, Brussels, Belgium, C/O GSK, Wavre, Belgium; 2 GSK, Rockville, Maryland, USA; 3 FISABIO Fundación para el Fomento Investigación Sanitaria y Biomédica de la Comunitat Valenciana, Valencia, Spain; 4 Hospital General de Durango, Durango, Mexico; 5 Department of Internal Medicine, National Taiwan University College of Medicine and National Taiwan University Hospital, Taipei, Taiwan; 6 Diagnostics Research Group, San Antonio, Texas, USA; 7 Service CIC, CHU Clermont-Ferrand, Clermont-Ferrand, France; 8 Servicio de Prevención, Hospital Clínico San Carlos, Madrid, Spain; 9 Linkou Chang Gung Memorial Hospital, Taoyuan City, Taiwan; 10 Korea University Guro Hospital, Seoul, Republic of Korea; 11 SoonChunhyang University Bucheon Hospital, Bucheon-si, Republic of Korea; 12 Clinical Trial Center, SU/Sahlgrenska Universitetssjukhuset, Göteborg, Sweden; 13 GSK, Rixensart, Belgium; 14 CHU de Québec-Université Laval, Québec City, Canada; 15 Praxisgemeinschaft Heimeranplatz, München, Germany; 16 Institute of Tropical Medicine, Universitätsklinikum Tübingen, Tübingen, Germany; 17 Colchester Research Group, Truro, Canada; 18 Division of Infectious Diseases, UBC, Island Health Authority, PerCuro Clinical Research Ltd., Victoria, Canada; 19 Taipei Veterans General Hospital, Taipei City, Taiwan; 20 National Yang Ming University School of Medicine, Taipei City, Taiwan; 21 Laboratório de Investigação Médica em Envelhecimento (LIM-66), Serviço de Geriatria, Hospital das Clínicas HCFMUSP, Faculdade de Medicina, Universidade de São Paulo, São Paulo, Brazil; 22 Department of Infectious Diseases, Hradec Kralove, Czechia; 23 Occupational Medicine and Family Medicine, China Medical University Hospital, Taichung, Taiwan; 24 Pori Vaccine Research Clinic, Vaccine Research Center, Tampere University, Pori, Finland; 25 Praxis Dr. med. Beate Moeckesch, Weinheim, Germany; 26 Ambulatório de Pesquisa Clínica, Santa Casa de Misericórdia de Belo Horizonte, Belo Horizonte, Brazil; 27 Korea University Ansan Hospital, Ansan, Republic of Korea; 28 Akademiska sjukhuset, Infektionskliniken, Uppsala, Sweden; 29 GSK, Wavre, Belgium; 30 UHZ Klinische Forschung, Essen, Germany; 31 Synexus Lancashire Clinical Research Centre, Chorley, United Kingdom; 32 Clinical Research Centre, Eskilstuna, Sweden; 33 Blekinge Institute of Technology, Department of Health, Karlstrona, Sweden; 34 KFB- Klinische Forschung Berlin, Berlin, Germany; 35 Institute of Laboratory Medicine and Vaccination Centre, Klinikum Würzburg Mitte, Standort Juliusspital, Würzburg, Germany; 36 Espoo Vaccine Research Clinic, Vaccine Research Center, Tampere University, Espoo, Finland; 37 Centro Paulista de Investigação Clínica, CEPIC, São Paulo, Brazil; 38 The Westmead Institute for Medical Research, Westmead, NSW, Australia; 39 University of Sydney, Sydney, NSW, Australia

**Keywords:** adjuvanted recombinant zoster vaccine, long-term efficacy, immune response persistence

## Abstract

**Background:**

This ongoing follow-up study evaluated the persistence of efficacy and immune responses for 6 additional years in adults vaccinated with the glycoprotein E (gE)-based adjuvanted recombinant zoster vaccine (RZV) at age ≥50 years in 2 pivotal efficacy trials (ZOE-50 and ZOE-70). The present interim analysis was performed after ≥2 additional years of follow-up (between 5.1 and 7.1 years [mean] post-vaccination) and includes partial data for year (Y) 8 post-vaccination.

**Methods:**

Annual assessments were performed for efficacy against herpes zoster (HZ) from Y6 post-vaccination and for anti-gE antibody concentrations and gE-specific CD4[2+] T-cell (expressing ≥2 of 4 assessed activation markers) frequencies from Y5 post-vaccination.

**Results:**

Of 7413 participants enrolled for the long-term efficacy assessment, 7277 (mean age at vaccination, 67.2 years), 813, and 108 were included in the cohorts evaluating efficacy, humoral immune responses, and cell-mediated immune responses, respectively. Efficacy of RZV against HZ through this interim analysis was 84.0% (95% confidence interval [CI], 75.9–89.8) from the start of this follow-up study and 90.9% (95% CI, 88.2–93.2) from vaccination in ZOE-50/70. Annual vaccine efficacy estimates were >84% for each year since vaccination and remained stable through this interim analysis. Anti-gE antibody geometric mean concentrations and median frequencies of gE-specific CD4[2+] T cells reached a plateau at approximately 6-fold above pre-vaccination levels.

**Conclusions:**

Efficacy against HZ and immune responses to RZV remained high, suggesting that the clinical benefit of RZV in older adults is sustained for at least 7 years post-vaccination.

**Clinical Trials Registration.** NCT02723773.

## Teaser

Efficacy against herpes zoster and immune responses to the adjuvanted recombinant zoster vaccine plateaued at high levels between 5.1 and 7.1 years (mean) post-vaccination, suggesting that its clinical benefit in older adults is sustained for at least 7 years post-vaccination.

Herpes zoster (HZ) is a disease caused by reactivation of the varicella-zoster virus (VZV), which establishes lifelong latency in the dorsal root ganglia after the initial infection. HZ occurs most frequently after the age of 50 years, and the risk of HZ increases further with age due to immunosenescence [1, 2]. HZ can have painful and debilitating complications, such as post-herpetic neuralgia [2], yet it is preventable by vaccination [3–5]. As vaccine-induced immunity can wane over time [6], it is critical to assess if vaccines induce long-lasting protective immunity. For instance, a single dose of the live-attenuated zoster vaccine (ZVL, Zostavax, Merck Sharp & Dohme Corp) was 68% effective against HZ in individuals aged ≥50 years during the first year post-vaccination, but its effectiveness waned to 32% by the eighth year post-vaccination [7]. Its efficacy against HZ in those aged ≥60 years also dropped from 62% in the first year post-vaccination to nonstatistically significant levels beyond the eighth year post-vaccination [8].

The adjuvanted recombinant zoster vaccine (RZV, Shingrix, GSK), consisting of recombinant varicella-zoster glycoprotein E (gE) and the AS01_B_ adjuvant system, and administered as a 2-dose schedule, demonstrated ≥90% efficacy against HZ in all age groups ≥50 years, which was maintained over a 3.2- and 3.7-year follow-up period in 2 pivotal phase 3 trials (ZOE-50 and ZOE-70, respectively) [4, 5]. While a correlate of protection has not been established to date, additional data have shown that immune responses to RZV plateau at a substantial level above baseline from the fourth year post-vaccination onward, and mathematical modeling predicts that immune responses will persist at least 20 years post-vaccination [9].

At approximately 5 years after the 2-dose vaccination course in ZOE-50/70, former study vaccinees were offered the possibility to enroll in a long-term follow-up (LTFU) study evaluating RZV’s efficacy, immunogenicity, and safety for 6 additional years. Here, we present the results of an interim analysis based on data collected after at least 2 of the 6 additional years of follow-up from this LTFU study.

## METHODS

### Study Design

**Figure 1.**
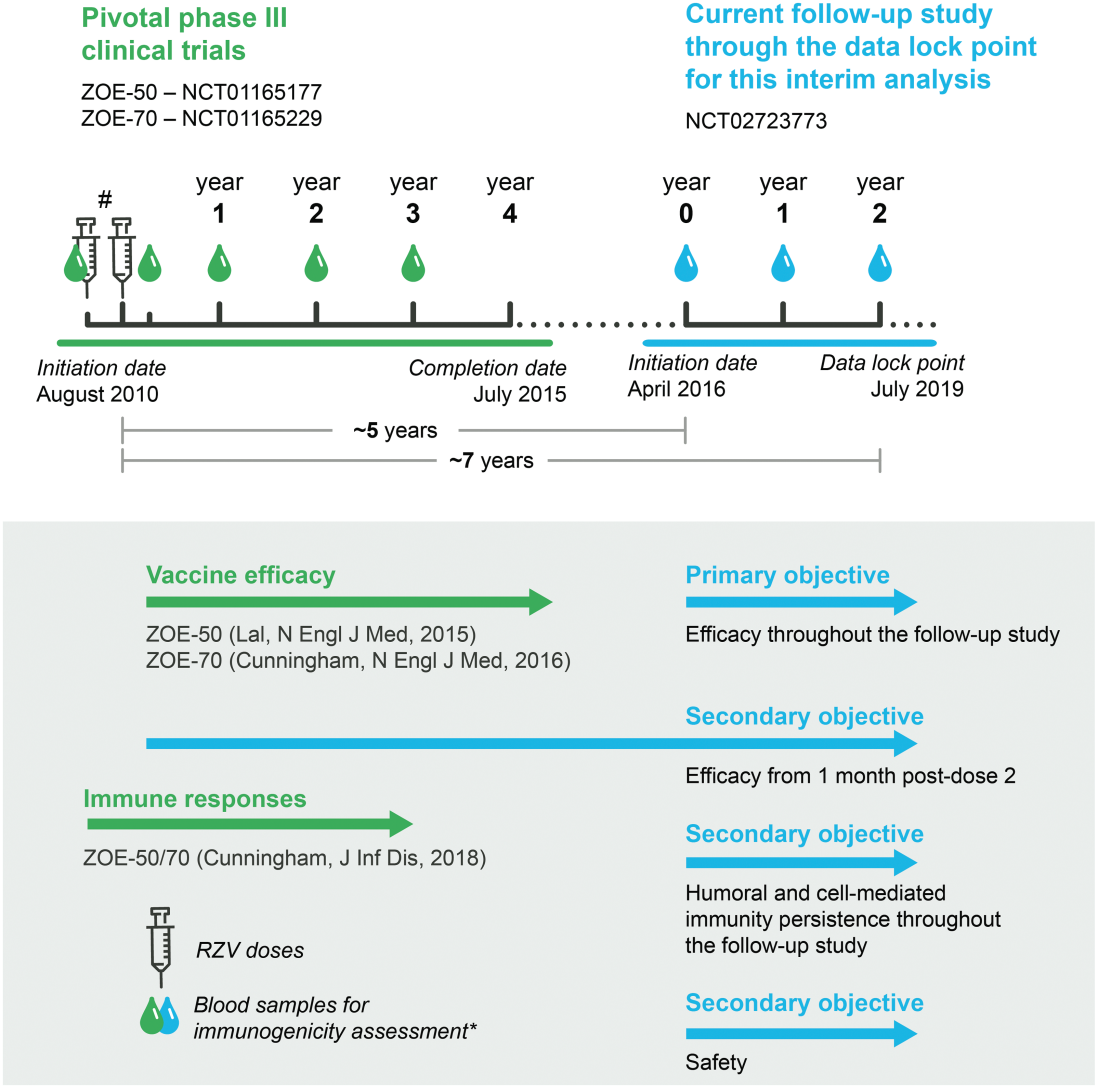
Study design of the pivotal phase 3 clinical trials (ZOE-50 and ZOE-70) and current long-term follow-up study. ^#^Pre-vaccination blood sample and 2 RZV doses 2 months apart. *Collected from subsets of participants for humoral and cell-mediated immunity assessment. Abbreviations: RZV, adjuvanted recombinant zoster vaccine; ZOE-50, pivotal phase 3 clinical trial of RZV in adults aged ≥50 years; ZOE-70, pivotal phase 3 clinical trial of RZV in adults aged ≥70 years.

This is an ongoing open-label, phase 3B, LTFU study of the pivotal phase 3 clinical trials with RZV (ZOE-50: NCT01165177 and ZOE-70: NCT01165229; approximately 30 000 participants), which is being conducted at 163 centers in 18 countries/regions (Australia, Brazil, Canada, Czech Republic, Estonia, Finland, France, Germany, Hong Kong, Italy, Japan, Republic of Korea, Mexico, Spain, Sweden, Taiwan, the United Kingdom, and the United States). The study was initiated on 16 April 2016, and the data lock point for the interim analysis presented herein was on 30 July 2019, when participants had completed at least 2 additional years of follow-up (Figure 1). The total follow-up period varied between participants, but data accrual through year (Y) 7 post-vaccination was complete at the data lock point. Since many study participants had already reached Y8 post-vaccination at the data lock point, we also present partial results for this time point.

All participants or their legally acceptable representatives provided written informed consent at enrollment. The study protocol was reviewed and approved by national, regional, or investigational center independent ethics committees or institutional review boards. The study was conducted in accordance with the Declaration of Helsinki and the Principles of Good Clinical Practice. The study is registered on ClinicalTrials.gov (NCT02723773).

In addition to the LTFU for RZV’s efficacy, immunogenicity, and safety, this study also aimed to evaluate the administration of 1 or 2 additional RZV doses, for which a subset of 240 participants were randomized 1:1:2 to receive 1 RZV dose, 2 RZV doses, or no treatment. The results for revaccination will be disclosed in a future publication.

**Figure 2.**
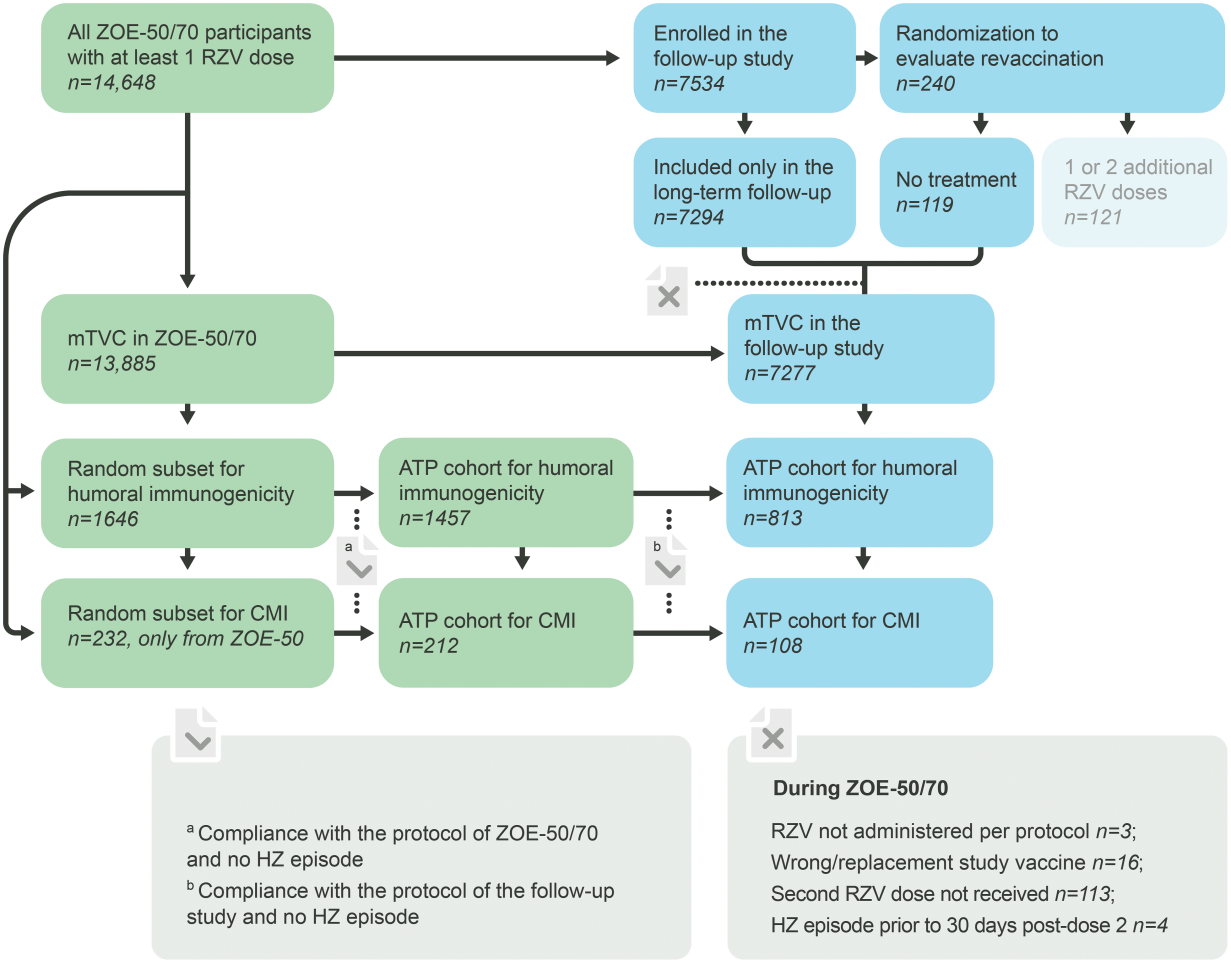
Study cohorts. Abbreviations: ATP, according-to-protocol; CMI, cell-mediated immunity; HZ, herpes zoster; mTVC, modified total vaccinated cohort; RZV, adjuvanted recombinant zoster vaccine; ZOE-50, pivotal phase 3 clinical trial of RZV in adults aged ≥50 years; ZOE-70, pivotal phase 3 clinical trial of RZV in adults aged ≥70 years.

Here, we present the interim results of the LTFU, which are based on study participants who did not receive additional RZV doses during this study, including those enrolled only for LTFU and the participants randomized to receive no treatment (Figure 2).

### Study Participants

The parent phase 3 trials (ZOE-50/7) enrolled adults aged ≥50 and ≥70 years, respectively, according to previously described inclusion/exclusion criteria [4, 5]. ZOE-50/70 participants were randomized 1:1 to receive 2 RZV or placebo doses, and participants who received at least 1 RZV dose were eligible for inclusion in this phase 3B LTFU study. The use of immunosuppressive and immune-modifying treatments and VZV or HZ vaccines other than RZV (administered during ZOE-50/70) were not allowed during the study. A complete list of inclusion and exclusion criteria is presented in Supplementary Materials 1.

### Study Vaccine

During the ZOE-50/70 studies, participants received 2 RZV doses, administered intramuscularly 2 months apart. RZV consists of 50 μg of recombinant VZV gE and the AS01_B_ adjuvant system (containing 50 μg of 3-O-desacyl-4’-monophosphoryl lipid A, 50 μg of *Quillaja saponaria* Molina, fraction 21 [licensed by GSK from Antigenics LLC, a wholly owned subsidiary of Agenus Inc, a Delaware, United States, corporation], and liposome) [10]. During the current LTFU study, no additional RZV doses were administered to participants included in the analyses presented herein.

### Outcomes and Assessments

#### Objectives

The primary objective of this study was to assess the efficacy of RZV in the prevention of HZ over the total duration of the current LTFU study (Figure 1). Secondary objectives included the assessment of RZV’s efficacy in preventing HZ from 1 month post-dose 2 (administered in the ZOE-50/70 studies) until the end of the current LTFU study overall and by year post-vaccination, persistence of humoral and cell-mediated immune (CMI) responses to RZV at each year post-vaccination, and safety.

#### Ascertainment of HZ cases

Suspected HZ was defined as a new unilateral rash accompanied by pain (broadly defined to include allodynia, pruritus, or other sensations) with no alternative diagnosis. HZ cases were confirmed following the procedures used in the ZOE-50/70 studies [4, 5]. Briefly, suspected HZ cases diagnosed clinically by the investigator were confirmed by polymerase chain reaction or, when not possible, by a HZ ascertainment committee (3–5 independent physicians with HZ expertise) based on the available clinical information (ie, rash and pain evaluations, digital photographs, and clinical progress notes).

#### Assessment of immunogenicity

Anti-gE antibody concentrations were measured by anti-gE enzyme-linked immunosorbent assay with a technical cutoff assay quantification of 97 milli-international units per milliliter (mIU/mL). Frequencies of gE-specific CD4[2+] T cells (CD4+ T cells expressing at least 2 of the 4 activation markers assessed: interferon-γ, interleukin-2, tumor necrosis factor-α, and CD40 ligand) were measured by intracellular cytokine staining and detection by flow cytometry after in vitro stimulation with a pool of peptides covering the gE ectodomain [11].

#### Assessment of safety

Serious adverse events (SAEs) related to study participation and HZ complications (ie, post-herpetic neuralgia, disseminated HZ, and similar complications) were collected and recorded. Post-herpetic neuralgia was defined as HZ-associated severe “worst” pain persisting or appearing >90 days post-rash onset. Disseminated HZ was defined as ≥6 HZ lesions outside the primary dermatome.

### Statistical Analyses

All analyses were descriptive, and <50% (6406) of evaluable RZV recipients from the ZOE-50/70 studies were expected to be enrolled in this LTFU study. The presumed approximately 6000 participants evaluable for efficacy would allow a lower limit for the annual vaccine efficacy estimate >30% to be achieved with a probability of ≥99%.

In the ZOE-50/70 studies and this LTFU study, efficacy was evaluated in the modified total vaccinated cohort (mTVC), restricted to participants who received both RZV doses in the ZOE-50/70 studies and did not develop a confirmed HZ episode before 1 month post-dose 2. As the follow-up of a ZOE-50/70 participant for HZ ended at the first confirmed HZ case, inclusion in the mTVC of the current LTFU study was also precluded by the occurrence of an HZ episode during the ZOE-50/70 studies. In the current study, the follow-up of a participant for HZ ended at the first confirmed HZ case, last contact date, or data lock point for the interim analysis. A complementary efficacy analysis will be performed at the end of the study on all participants who received at least 1 RZV dose.

In the ZOE-50/70 studies and this LTFU study, humoral and CMI responses were assessed in subsets of the entire study population. The persistence of humoral/CMI responses in this LTFU study was evaluated in the according-to-protocol (ATP) cohort for persistence, which included all evaluable participants from the ATP cohort for immunogenicity in the ZOE-50/70 trials who complied with the protocol of the current LTFU study, had immunogenicity results available at the considered time point, and did not report a condition that may confound immunogenicity results, including HZ and malignancy, up to the time point considered (Figure 2). Long-term safety was evaluated in participants who were included only in the LTFU.

After demonstrating >90% efficacy of RZV in the ZOE-50/70 trials, 8687 placebo recipients from ZOE-50/70 were also vaccinated with RZV in a separate study [12]. Hence, in the absence of an unvaccinated placebo group for the LTFU, the efficacy analyses (overall and annual) for the period of this LTFU study used historical control estimates from the ZOE-50/70 placebo groups recorded during the trials, adjusted for age and region at randomization during the ZOE-50/70 studies. Vaccine efficacy was calculated, and 95% confidence intervals (CIs) were estimated based on the variance of the observed population in this study and treating the historical control as constant. The annual estimates for Y1–Y4 were calculated using the actual incidence rates from the RZV and placebo groups of the ZOE-50/70 studies. For the pooled overall vaccine efficacy analysis (ZOE-50/70 + current LTFU study), the annual efficacy results from the ZOE-50/70 studies were combined with the annual estimates from the current LTFU study, and the overall efficacy and asymptotic CI were calculated giving equal weight to each annual estimate.

The anti-gE antibody geometric mean concentration (GMC) calculations were performed by taking the anti-log of the mean of the log concentration transformations. For GMC calculation, concentrations below the cutoff of the assay were given an arbitrary value equal to half the cutoff.

The frequency of gE-specific CD4[2+] T cells was calculated as the difference between the frequency of CD4[2+] T cells stimulated in vitro with gE peptides and those stimulated with culture medium alone. The results were expressed as the number of CD4[2+] T cells per 10^6^ total T cells and tabulated as descriptive statistics (minimum, first quartile, median, third quartile, maximum).

The Power law model [9], a linear mixed-effect model in which the log of the response was regressed on the log of the time, was used to model the log_10_-transformed anti-gE antibody GMCs and the geometric mean frequency of CD4[2+] T cells over time. The covariates included the log-transformed pre-vaccination response and the annual frame post-vaccination. Available annual data from Y1–Y8 post-vaccination for participants included in the LTFU of this study were used to fit the model. For Y1–Y3, annual ZOE-50/70 data were used. For Y4, data were not available due to the gap between the ZOE-50/70 and the present LTFU study (see Figure 1). Statistical analyses were performed using the SAS Drug Development system (SAS Institute Inc, Cary, NC, USA).

## RESULTS

### Study Participants

Of the 14 648 ZOE-50/70 participants who received at least 1 RZV dose [4, 5], 7413 (50.6%) were enrolled for the long-term efficacy assessment, exceeding the expected sample size. Of these, 7277 had previously received both RZV doses and were included in the mTVC for the efficacy assessments, 813 in the ATP cohort for humoral immunity persistence, and 108 in the ATP cohort for CMI persistence (Figure 2). In the mTVC, the mean age at first vaccination in the ZOE-50/70 studies was 67.2 (±9.4) years (Table 1); 60.7% were women, and 76.5% of participants were of European ancestry. Demographic characteristics in the ATP cohorts for humoral and CMI persistence were similar to those in the mTVC (Table 1).

**Table 1.** Summary of Demographic Characteristics

Characteristic	Parameter or Category	Modified Total Vaccinated Cohort, N = 7277	According-to-Protocol Subset for Humoral Immunity Persistence, N = 813	According-to-Protocol Subset for Cell-Mediated Immunity Persistence, N = 108
Age at first vaccination in ZOE-50 and ZOE-70, years	Mean ± standard deviation	67.2 ± 9.4	66.1 ± 9.0	62.6 ± 8.2
Sex, n (%)	Female	4419 (60.7)	494 (60.8)	56 (51.9)
	Male	2858 (39.3)	319 (39.2)	52 (48.1)
Ethnicity, n (%)	American Hispanic or Latino	529 (7.3)	53 (6.5)	0 (0.0)
	Not American Hispanic or Latino	6748 (92.7)	760 (93.5)	108 (100)
Geographic ancestry,^a^ n (%)	European	5567 (76.5)	582 (71.5)	75 (69.4)
	Asian	1354 (18.7)	207 (25.5)	31 (28.7)
	African	62 (0.9)	8 (1.0)	2 (1.9)
	Other	294 (4.0)	16 (2.0)	0 (0.0)

N is the total number of participants; n (%) is the number (percentage) of participants in a given category.

Abbreviations: ZOE-50, pivotal phase 3 clinical trial of RZV in adults aged ≥50 years; ZOE-70, pivotal phase 3 clinical trial of RZV in adults aged ≥70 years.

^a^European ancestry: Caucasian/European Heritage or Arabic/North African Heritage; Asian ancestry: Central/South Asian Heritage or East Asian Heritage or Japanese Heritage or Southeast Asian Heritage; African ancestry: African Heritage/African American; Other ancestry: American Indian or Alaskan Native, Native Hawaiian or Other Pacific Islander, or Other.

### Long-Term Efficacy

During the period ranging from a mean of approximately 5.1 to approximately 7.1 years post-vaccination, 27 and 169 confirmed HZ cases occurred in the vaccine and control groups, respectively, and thus RZV was 84.0% (95% CI, 75.9–89.8) efficacious in preventing HZ (Table 2). Through the entire post-vaccination follow-up period, ranging from 1 month post-dose 2 to a mean of approximately 7.1 years post-vaccination, 59 and 651 confirmed HZ cases occurred in the vaccine and control groups, respectively, and thus efficacy of RZV against HZ was 90.9% (95% CI, 88.2–93.2). Annual vaccine efficacy estimates reached a plateau >84% between Y4 and Y6 post-vaccination (Table 2).

**Table 2.** Vaccine Efficacy in the ZOE-50 and ZOE-70 Studies and the Current Long-Term Follow-up Study After at Least 2 Additional Years of Follow-up

	Adjuvanted Recombinant Zoster Vaccine				Historical Control^a^/Placebo Group in ZOE-50 and ZOE-70^b^				Vaccine Efficacy, % (95% Confidence Interval)
	N	n	Sum of Follow-up Years	Incidence (per 1000 Person-Years)	N	n	Sum of Follow-up Years	Incidence (per 1000 Person-Years)	
Vaccine efficacy in the current follow-up study: primary objective (up to the data lock point for the interim analysis in the current follow-up study)									
Overall^a^	7277	27	19 621.7	1.4	7277	169	19 621.7	8.6	84.0 (75.9–89.8)
Vaccine efficacy from 1 month post-dose 2: secondary objective (up to the data lock point for the interim analysis in the current follow-up study)									
Overall	13 881	59	72 744.6	0.8	13 881	651	72 744.6	8.9	90.9 (88.2–93.2)
Year 1^b^	13 881	3	13 744.5	0.2	14 035	130	13 823.3	9.4	97.7 (93.1–99.5)
Year 2^b^	13 569	10	13 415.6	0.7	13 564	136	13 332.5	10.2	92.7 (86.2–96.6)
Year 3^b^	13 185	9	13 016.1	0.7	13 074	116	12 834.0	9.0	92.4 (85.0–96.6)
Year 4^b^	12 757	10	12 946.7	0.8	12 517	95	12 637.4	7.5	89.8 (80.3–95.2)
Year 6^a^	7277	10	7208.8	1.4	7277	66	7208.8	9.2	84.9 (70.4–93.1)
Year 7^a^	7097	10	6993.1	1.4	7097	68	6993.1	9.7	85.3 (71.3–93.3)
Year 8 ^a^^,^^c^	6876	7	5160.2	1.4	6876	44	5160.2	8.5	84.1 (64.4–94.0)

No data are available for year 5 because that period corresponds to the gap between ZOE-50 and ZOE-70 and the current follow-up study. The follow-up ceased at the first occurrence of a confirmed herpes zoster (HZ) episode. N is the number of individuals included in each group; n is the number of individuals having at least 1 confirmed herpes zoster episode.

Abbreviations: ZOE-50, pivotal phase 3 clinical trial of RZV in adults aged ≥50 years; ZOE-70, pivotal phase 3 clinical trial of RZV in adults aged ≥70 years.

^a^Adjuvanted recombinant zoster vaccine (RZV) vs matched historical controls from the placebo group in the ZOE-50/70 studies, adjusted for age and region at randomization during the ZOE-50/70 studies. The same N and follow-up period were considered for the historical control and vaccinated group. n for historical controls represents the projected number of included placebo group participants from ZOE-50/70 with at least 1 confirmed HZ episode based on the estimated incidence rate.

^b^RZV vs placebo recipients from the ZOE-50/70 trials adjusted for region.

^c^At the data lock point for the interim analysis in the current follow-up study, data collection for year 8 was still incomplete.

*P* < .0001 for all vaccine efficacy estimates.

### Immunogenicity Persistence

**Figure 3.**
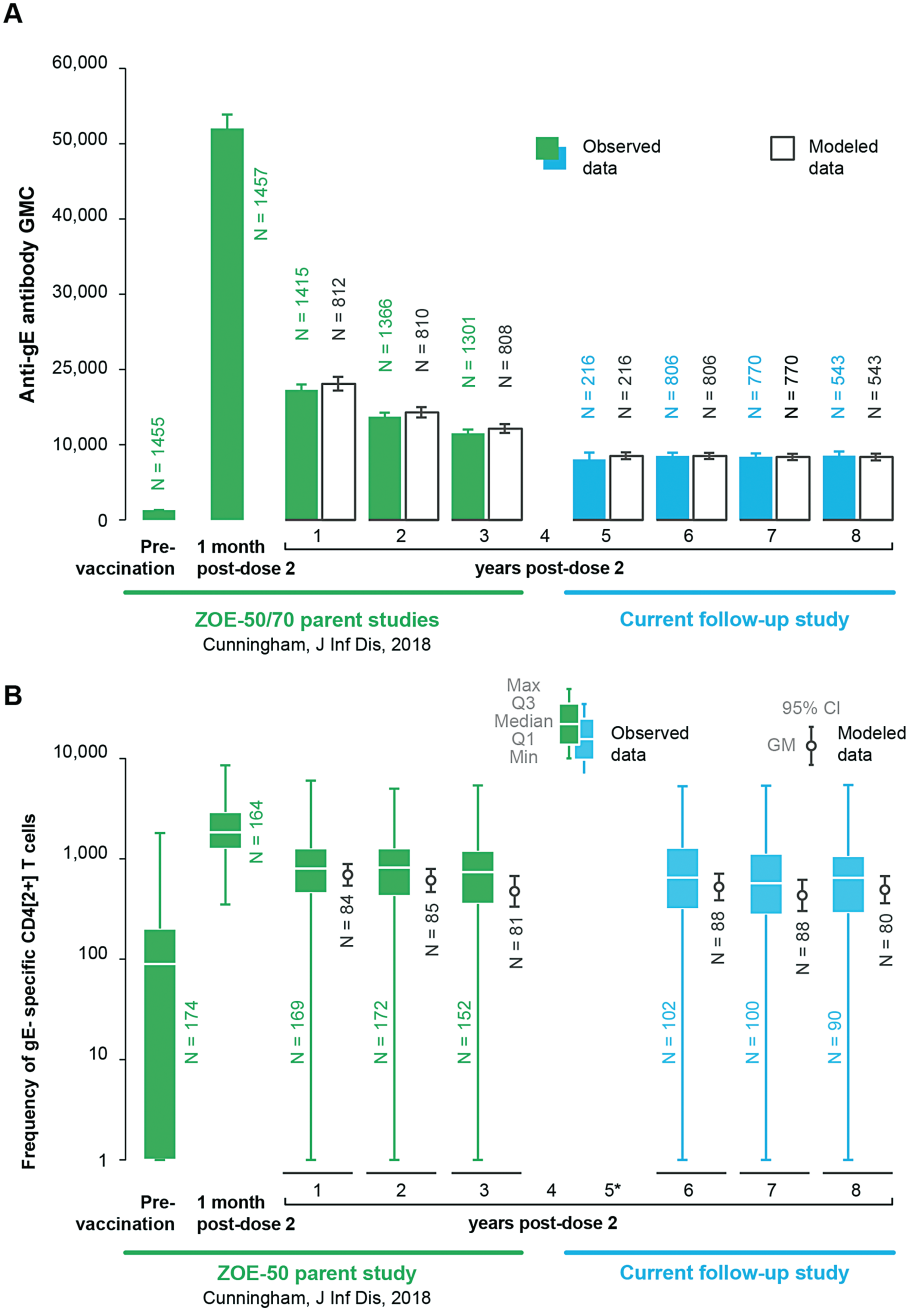
Persistence of humoral and cell-mediated immune responses to RZV in the ZOE-50 and ZOE-70 studies and the current follow-up study up to the data lock point for the interim analysis (according-to-protocol cohort for immunogenicity persistence). *A,* Anti-gE antibody GMCs. *B,* Frequencies of CD4[2+] T cells. *Data not shown because only 3 participants had available results for this analysis. Abbreviations: CD4[2+] T cells, CD4 T cells expressing at least 2 of 4 assessed activation markers: interferon-γ, interleukin-2, tumor necrosis factor-α, and CD40 ligand; Q1 and Q3, first and third quartiles, respectively; CI, confidence interval; gE, glycoprotein E; GM, geometric mean; GMC, geometric mean concentration; N, number of participants with available results; RZV, adjuvanted recombinant zoster vaccine; ZOE-50, pivotal phase 3 clinical trial of RZV in adults aged ≥50 years; ZOE-70, pivotal phase 3 clinical trial of RZV in adults aged ≥70 years.

The anti-gE antibody GMC was 1320.5 (95% CI, 1253.6–1391.0) mIU/mL pre-vaccination, 17 296.9 (95% CI, 16 614.7– 18 007.1) mIU/mL at Y1 (in ZOE-50/70), 8053.5 (95% CI, 7239.3–8959.4) mIU/mL at Y5, and plateaued thereafter at >6-fold over the pre-vaccination level (Figure 3A). The median CD4[2+] T-cell frequency was 89.8 (interquartile range [IQR], 1.0–202.4) pre-vaccination, 799.9 (IQR, 454.3–1277.3) at Y1 (in ZOE-50/70), 652.4 (IQR, 314.3–1293.0) at Y6, and plateaued thereafter at >6-fold over the pre-vaccination level (Figure 3B). Similar kinetics resulted by mathematical modeling (Figure 3).

### Long-Term Safety

No deaths or other SAEs were considered causally related to vaccination. Two participants with confirmed HZ reported HZ-related complications during the LTFU study. At 6 years and 4 months post-vaccination, in a participant aged 88 years, an HZ episode involving dermatome C2 (resolved in 55 days) was complicated by post-herpetic neuralgia. Although slowly resolving, it was still ongoing at the data lock point. At 7 years and 3 months post-vaccination, a participant aged 80 years experienced disseminated HZ of moderate intensity involving dermatomes L2–L5 posteriorly and dermatomes T10–T11 anteriorly, which resolved in 40 days.

## DISCUSSION

**Figure 4.**
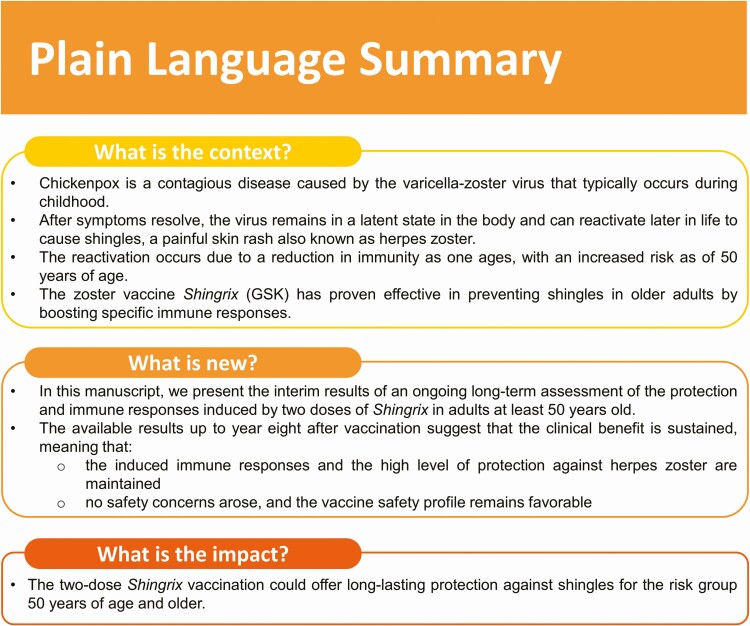
Plain language summary. Abbreviation: GSK, GlaxoSmithKline.

Almost all adults are infected with VZV and consequently remain at risk for HZ and potentially severe complications throughout their lifetime [13]. Reactivation of VZV primarily occurs in adults aged ≥50 years, and the risk continues to increase with age [1, 2]. In the absence of HZ vaccination, approximately half of individuals aged >85 years will have had at least 1 HZ episode in their lifetime [14]. This persistent and increasing risk requires a vaccine that offers long-term protection. The current LTFU study of >7000 participants vaccinated at a mean age of 67.2 years in 2 pivotal clinical trials with RZV showed that the efficacy against HZ remained high through Y7 post-vaccination. A plain language summary contextualizing the results and their relevance to and impact on potential clinical research is displayed in Figure 4.

Our study’s primary analysis showed an overall efficacy of RZV in preventing HZ of 84.0% for the period ranging between a mean of 5.1 and 7.1 years post-vaccination in the ZOE-50/70 studies. From vaccination through a mean of 7.1 years post-vaccination, efficacy was 90.9%. Annual estimates reached a plateau by Y6, which remained at >84% up to Y8 post-vaccination when the final assessment in this interim analysis was made. In contrast, in adults aged ≥60 years at vaccination, the efficacy of ZVL became nonstatistically significant beyond the eighth year post-vaccination [8]. Additionally, the effectiveness of ZVL against HZ in those aged ≥50 years decreased from 67.5% in the first year to 31.8% in the eighth year post-vaccination [7].

In the ZOE-50/70 studies, humoral and CMI responses peaked at 1 month post-dose 2, declined rapidly through Y1, and declined slowly or plateaued from Y1 through Y3 post-vaccination when the last immunogenicity assessment was made [11]. Throughout the current LTFU study of former ZOE-50/70 participants, both humoral and CMI responses plateaued at approximately 6-fold above pre-vaccination levels. This is consistent with previous observations in adults vaccinated at the age of ≥60 years, in whom immune responses to RZV reached a plateau at Y4 post-vaccination and were maintained at essentially constant levels through the last assessment at Y10 post-vaccination [9]. In the same study evaluating RZV persistence in adults vaccinated at the age of ≥60 years, 3 mathematical models predicted persistence of humoral and CMI responses through at least 20 years post-vaccination [9]. We used the best-fitted of these 3 models (based on the Akaike information criterion [15]), that is, the Power law model, to model immune responses in our LTFU study of ZOE-50/70 participants vaccinated at age ≥50 years and found that modeled immune responses were in close agreement with those observed.

Our results have some limitations but also some strengths. Almost half of the former ZOE-50/70 participants did not enroll in this LTFU study. However, this is likely due to the age of former ZOE-50/70 participants (mean age >67 years at vaccination in ZOE-50/70) and the time between vaccination in ZOE-50/70 and the start of this follow-up study (5 years). At the data lock point for the present interim analysis, not all participants had reached Y8 post-vaccination, and since the follow-up is ongoing, precision of the vaccine efficacy estimate and the sample size for immunogenicity assessments for this time point will increase in subsequent analyses. Participants will be followed through 11 years post-vaccination, and final data will confirm whether the observed plateaus in immune responses and vaccine efficacy persist. While HZ cases that occurred during the ZOE-50/70 and this LTFU study were ascertained according to the same strict protocol, those that occurred during the 1-year gap between the ZOE-50/70 and this LTFU study were only recorded at enrollment, without being confirmed per the same protocol. Therefore, vaccine efficacy for Y5 could not be estimated. This LTFU study did not have a concomitant placebo group, as placebo recipients from ZOE-50/70 had received RZV in a subsequent study [12]. Hence, we used HZ incidences from the placebo group of the ZOE-50/70 studies (adjusted for age and region at randomization in ZOE-50/70) to calculate vaccine efficacy in the present LTFU study. While previous long-term immunogenicity data were generated in a population of approximately 70 White adults vaccinated at age ≥60 years [9], our immunogenicity data were generated along with efficacy data on a larger and racially more heterogeneous population that also included adults aged 50–59 years at vaccination, suggesting that our results may be generalized to a broader population.

## CONCLUSIONS

This LTFU study of >7000 former participants of the ZOE-50/70 pivotal clinical trials with RZV shows that the efficacy against HZ remained high (84%) from approximately 5 to 7 years after the 2-dose vaccination course administered at the age of ≥50 (mean, 67.2) years. Annual vaccine efficacy estimates were >84% for each year since vaccination and were stable during this follow-up study through the interim analysis. Both humoral and CMI responses to RZV also plateaued throughout this LTFU, at >6-fold above pre-vaccination levels. Our findings suggest that the clinical benefit of RZV in older adults is sustained for at least 7 years post-vaccination.

## Supplementary Data

Supplementary materials are available at *Clinical Infectious Diseases* online. Consisting of data provided by the authors to benefit the reader, the posted materials are not copyedited and are the sole responsibility of the authors, so questions or comments should be addressed to the corresponding author.

ciab629_suppl_Supplementary_Material

## Notes

***Author Contributions.*** Conceptualization: C. B., H. S. C., C. J. Y., and T. Z. Validation: C. B., E. D. P., J. D. D., A. H., P. P., B. S., A. S., M. S., J. C. T., C. J. Y., and T. Z. Formal analysis: M. S. and T. Z. Investigation: C. A., J. B., C. C., H. S. C., H. J. C., E. J. C., A. L. C., J. D. D., M. D., M. E., M. F., W. G., S. J. H., T. J. A. D. S., P. K., C. S. L., J. M., B. M., C. M. D. O., D. W. P., K. P., G. P., C. P., L. R., J. S. B., I. S., T. S., J. C. T., B. U., C. J. Y., and C. Z. Resources: H. S. C., E. D. P., S. J. H., C. S. L., D. W. P., P. P., G. P., B. S., M. S., C. J. Y., T. Z., and C. Z. Writing (original draft): C. B. and A. H. Visualization: C. B., A. L. C., A. H., S. J. H., B. S., A. S., M. S., and T. Z. Supervision: C. B., A. H., and A.S. All authors contributed to the writing (review and editing) of the manuscript and approved the final, submitted version.

***Acknowledgments.*** The authors thank all study participants, the clinical investigators and study staff, and the entire GSK team involved in the ZOE-50, ZOE-70, and Zoster-049 studies. The authors also thank the Modis platform, on behalf of GSK, for editorial assistance and manuscript coordination. Alpár Pöllnitz provided medical writing support, and Sander Hulsmans coordinated the manuscript development and provided editorial support.

Zoster-049 nonauthor collaborators (Study Group; in alphabetical order):

Michael Adams, Anitta Ahonen, Eugene Athan, Jose-Fernando Barba-Gómez, Piero Barbanti, Elisabeth Barberan, Alain Baty, Niklas Bengtsson, Juergen Berger-Roscher, Katarina Berndtsson Blom, Loïc Boucher, Alain Boye, François Brault, Laurie Breger, Carles Brotons Cuixart, Christine Cerna, Clóvis Cunha, Benoit Daguzan, Antje Dahmen, Susan Datta, Maria Giuseppina Desole, Petr Dite, Jan Dutz, John Earl, William Ellison, Jurij Eremenko, Takashi Eto, Xavier Farrés Fabré, Cecil Farrington, Pierre André Ferrand, Matthew Finneran, David Francyk, Marshall Freedman, George Freeman, Peter Gal, Jean-Sebastien Gauthier, Beatrice Gerlach, Iris Gorfinkel, Christine Grigat, Josef Grosskopf, Monika Hamann, Pascal Hanrion, Paul Hartley, Ken Heaton, Agnes Himpel-Boenninghoff, Thomas Horacek, David Shu Cheong Hui, Yieng Huong, Giancarlo Icardi, Gabriele Illies, Junya Irimajiri, Alen Jambrecina, Hyo Youl Kim, Christiane Klein, Uwe Kleinecke, Hans-Joachim Koenig, Satu Kokko, Pekka Koskinen, Maximilian Kropp, Rie Kuroki, Outi Laajalahti, Pierre Lachance, Jacob Lee, Jin-Soo Lee, Peter Levins, Robert Lipetz, Bo Liu, Martin Lundvall, Mary Beth Manning, Frederick Martin, Pyrene Martínez Piera, Damien McNally, Shelly McNeil, Guglielmo Migliorino, Stephan Morscher, Michael Mueller, Abul Kashem Munir, Kenjiro Nakamura, Silvia Narejos Pérez, Yuji Naritomi, Patrice Nault, José Luiz Neto, Concepción Núñez López, Hiroaki Ogata, Åke Olsson, Pauliina Paavola, Janice Patrick, Mercè Pérez Vera, Airi Poder, Terry Poling, Samir Purnell-Mullick, George Raad, Michael Redmond, Philippe Remaud, Ernie Riffer, Patrick Robert, Alex Rodríguez Badia, Maria Luisa Rodríguez de la Pinta, Robert Rosen, Shari Rozen, Dominique Saillard, Joachim Sauter, Axel Schaefer, Juergen Schmidt, Bernhard Schmitt, Christian Schubert, Ilkka Seppa, Edmund Kwok Yiu Sha, Gerald Shockey, Sylvia Shoffner, Elina Sirnela-Rif, Tommaso Staniscia, Hirohiko Sueki, Shin Suzuki, Denis Taminau, Guy Tellier, Manuel Terns Riera, Azhar Toma, Nicole Toursarkissian, Mark Turner, Anna Vilella Morató, Juergen Wachter, Brian Webster, Karl Wilhelm, Jonathan Wilson, Wilfred Yeo, Irina Zahharova.

***Disclaimer.*** Shingrix is a trademark owned by or licensed to GSK. Zostavax is a trademark of Merck Sharp & Dohme Corp.

***Financial support.*** GlaxoSmithKline Biologicals SA sponsored this work in all stages of the study conduct and analysis. GlaxoSmithKline Biologicals SA also took responsibility for all costs associated with developing and publishing the present manuscript.

***Potential conflicts of interest.*** E. D. P., A. H., P. P., B. S., A. S., M. S., and T. Z. were employees of the GSK group of companies (GSK) when the study was designed, initiated, and/or conducted. E. D. P., A. S., B. S., and T. Z. declare they hold shares in GSK as part of their remuneration. C. B. is a paid consultant at Aixial, an Alten Company, serving as a clinical research and development lead on behalf of GSK. C. A., J. B., M. E., and C. Z. declare grants from GSK paid to their institution/company, and L. R. reports salary payments from Region Sormland to the study nurses during the conduct of the study. Outside the submitted work, J. D. D. declares research grants paid to his institution by GSK and MSD, and he partook in advisory boards organized by GSK and MSD; M. E. reports grants from DFG, EDCTP, EKFS, and DZIF; A. L. C. received a grant from GSK and honoraria from Sequirus paid to his institution; M. F. previously received consulting fees from GSK unrelated to the authorship of any materials in this study or any other; and T. S. reports personal fees from GSK, Pfizer, Sanofi, Biogen, Merck-Serono, Biotest, and Sequirus. All other authors report no potential conflicts. The authors declare no other non-financial relationships and activities or conflicts of interest.

***Data sharing statement.*** The interim data analyzed during the current study are not available on a public registry but are available from the corresponding author on reasonable request.
