# Supplementary material for: The Adjuvanted Recombinant Zoster Vaccine Confers Long-Term Protection Against Herpes Zoster: Interim Results of an Extension Study of the Pivotal Phase 3 Clinical Trials ZOE-50 and ZOE-70
Source: Clin Infect Dis. 2021 Jul 20;74(8):1459–67. doi: 10.1093/cid/ciab629 (PMC9049256; doi:10.1093/cid/ciab629)
Supplement: ciab629_suppl_Supplementary_Material [file ciab629_suppl_supplementary_material.docx]

**The Adjuvanted Recombinant Zoster Vaccine Confers Long-term Protection Against Herpes Zoster: Interim Results of an Extension Study of the Pivotal Phase III Clinical Trials (ZOE-50 and ZOE-70)**

# Supplementary material

# Supplemental Text 1. Inclusion/exclusion criteria

# Inclusion criteria:

(I) Individuals who, in the opinion of the investigator, could and would comply with the requirements of the protocol (e.g., completion of the diary cards, return for follow-up visits, ability to have scheduled contacts to allow evaluation during the study), or (II) individuals with a caregiver who, in the opinion of the investigator, could and would comply with the requirements of the protocol (e.g., completion of the diary cards, availability for follow-up contacts)

Written informed consent obtained from the participant/legally acceptable representative prior to performance of any study-specific procedure

Individuals who participated in ZOE-50 or ZOE-70 studies and received at least one adjuvanted recombinant zoster vaccine (RZV) dose.

*Additional inclusion criteria for participants randomized to evaluate additional RZV dosing in this long-term follow-up study*:

Women of non-childbearing potential could be enrolled. Non-childbearing potential was defined as pre-menarche, current tubal ligation, hysterectomy, ovariectomy, or post-menopause.

Women of childbearing potential could have been enrolled if they had practiced adequate contraception for 30 days prior to study vaccination (i.e., additional RZV dose) and had a negative pregnancy test on the day of vaccination, and had agreed to continue adequate contraception during the entire treatment period and for two months after completion of the additional RZV series.

# Exclusion criteria:

Use of any investigational or non-registered product (pharmaceutical product or device) at the time of enrollment or planned use during the study period.

Previous vaccination against varicella-zoster virus (VZV) or herpes zoster (HZ) and/or planned administration during the study of a VZV or HZ vaccine (including an investigational or non-registered vaccine other than RZV administered in the ZOE-50/70 studies.

Chronic administration (defined as >14 consecutive days in total) of immunosuppressants or other immune-modifying drugs during the period starting six months prior to the first visit of this follow-up study or expected administration at any time during the study period. For corticosteroids, this meant prednisone ≥20 mg/day or equivalent. A prednisone dose of <20 mg/day was allowed. Inhaled, topical, and intra-articular corticosteroids were allowed.

Administration of long-acting immune-modifying drugs (e.g., infliximab, rituximab) within six months prior to the first visit of this follow-up study or expected administration at any time during the study period.

Any confirmed or suspected immunosuppressive or immunodeficient condition resulting from disease (e.g., malignancy, human immunodeficiency virus [HIV] infection) or immunosuppressive/cytotoxic therapy (e.g., medications used during cancer chemotherapy, organ transplantation, or to treat autoimmune disorders).

Administration of immunoglobulins and/or any blood products within three months prior to the first visit of this follow-up study or planned administration during the study period.

Prolonged use (>14 consecutive days) of oral and/or parenteral antiviral agents that are active against VZV (acyclovir, valacyclovir, famciclovir, etc.) and planned to be used during the study period for an indication other than to treat suspected or confirmed HZ or an HZ-related complication (topical use of these antiviral agents was allowed).

Significant underlying illness that, in the opinion of the investigator, would have been expected to interfere significantly during the study.

*Additional exclusion criteria for participants randomized to evaluate additional RZV dosing in this long-term follow-up study*:

Individuals who experienced a serious adverse event from first vaccination in the ZOE-50/70 studies to enrollment in this follow-up study that was considered related to study vaccine by either the investigator or the sponsor.

Individuals with a new onset of a potential immune-mediated disease (pIMD) or exacerbation of a pIMD from first vaccination in the ZOE-50/70 studies to enrollment in the current follow-up study.

Use of any investigational or non-registered product (pharmaceutical product or device) within 30 days preceding the first study vaccination in this follow-up study or planned use during the study period.

Administration or planned administration of any other immunizations within 30 days before the first study vaccination or scheduled within 30 days after study vaccination. However, licensed non-replicating vaccines (i.e., inactivated and subunit vaccines, including inactivated and subunit influenza vaccines for seasonal or pandemic flu, with or without adjuvant) could be administered up to 8 days prior to each additional RZV dose and/or at least 14 days after any dose.

History of allergic disease or reactions likely to be exacerbated by any component of the study vaccine. Additionally, allergic reactions to other material or equipment related to study participation (such as materials that may contain latex: gloves, syringes, etc. [the vaccine and vials in this study do not contain latex]) were also to be considered.

Pregnant or lactating woman.

Woman planning to become pregnant or planning to discontinue contraceptive precautions (if of childbearing potential).

Previous episode/history of HZ
